# Polymeric Micelles: A Promising Pathway for Dermal Drug Delivery

**DOI:** 10.3390/ma14237278

**Published:** 2021-11-28

**Authors:** Ana Parra, Ivana Jarak, Ana Santos, Francisco Veiga, Ana Figueiras

**Affiliations:** 1 Univ. of Coimbra, Department of Pharmaceutical Technology, Faculty of Pharmacy, Azinhaga de Santa Comba, Pólo III-Pólo das Ciências da Saúde, 3000-548 Coimbra, Portugal; anaparra.micf@gmail.com (A.P.); jarak.ivana@gmail.com (I.J.); anaicfsantos@gmail.com (A.S.); fveiga@ff.uc.pt (F.V.); 2 Univ. of Coimbra, REQUIMTE/LAQV, Group of Pharmaceutical Technology, Faculty of Pharmacy, Azinhaga de Santa Comba, Pólo III-Pólo das Ciências da Saúde, 3000-548 Coimbra, Portugal

**Keywords:** bioavailability, cosmetics, cutaneous application, efficacy, nanotechnology, polymeric micelles, safety, skin, skin disease

## Abstract

Nanotechnology is an area in great development and with application in the most varied fields of science, including cosmetic and pharmaceutical industries. Because conventional formulations for topical application are not always able to effectively penetrate the physical barrier that human skin exerts against factors and compounds of the external environment, polymeric micelles appear as alternative carriers for drugs and active ingredients delivery, also allowing ingredients with lower solubility and higher lipophilicity to be delivered. In fact, the augmented bioavailability of drugs, greater efficacy even at a lower dose, and selective drug delivery in specific organelles are very interesting advantages of the polymeric micelles usage in cutaneous application. As a consequence, they show a reduction in many of the local and systemic adverse effects, which might lead to an increase in patient compliance to the therapeutics, constituting a promising alternative to conventional topical formulations.

## 1. Introduction

Generally, skin is preferred by physicians and patients as a pathway for drug delivery in most dermatological treatments, as it is more accessible and avoids some systemic adverse effects, while providing a high drug concentration at the site of action. Despite the advantages of treating patients through their skin, there are some challenges to this type of formulations, as this dynamic human organ has a multilayer structure with a barrier function that constitutes an obstacle to the penetration and release of active substances. For instance, conventional cream formulations often offer a bioavailability that does not exceed 1–2% of the applied dose [1,2,3,4].

The prefix ‘nano’ is a derivation from a Greek prefix referring to something very small and represents one-thousand-millionth of a meter (10^−9^ m). Nanoscience studies these tiny particles, that are afterwards applied in nanotechnology, an area of technology present in several fields of industry and expertise, including physics, chemistry, biology, engineering, medicine, and pharmaceuticals sciences [5]. Therefore, nanotechnology is nowadays one of the most interesting resources in drug-delivery systems, and represents an alternative to skin barrier disrupting methods [6], using nanomaterials, defined by the European Commission as materials with at least one dimension in the nanoscale range, usually 1–100 nm [7], while solving some of the issues faced when it comes to topical drug formulations and efficacy [8]. 

Polymeric micelles (PM) have been widely studied since the 1990s [9], and even the late 1980s [10], for the development of self-assembled nanocarrier systems for delivering various bioactive molecules for cancer diagnosis and treatment, such as nucleic acids, reporter molecules, and cytostatic agents, which resulted in several formulations of approved drugs for clinical practice, mostly through intravenous infusion for chemotherapy. Theoretically, PM might solve various obstacles of conventional marketed drugs, including poor bioavailability, adverse side effects due to high-dose requirements, non-specific targeting, and low therapeutical index [11,12]. Since PMs have a great loading capacity, solubilization power, and stability in the blood stream they have been showing promising potential in the studies conducted as a possibility for higher efficacy with a superior safety profile [13,14]. Due to the advances in the medical imaging techniques, it is possible to accomplish diagnosis, therapy, and prognosis of a certain disease with a single micellar drug-delivery system [15]. The PMs’ difficult characterization, although, especially regarding interactions with the biological environment and their dynamics, is still a topic needing further analysis and discussion [8]. Another aspect that presents a hurdle to clinical translation of polymer-based nanomicelles is the scalable and reproducible manufacturing process of both copolymers and drug-loaded micelles [16,17]. Since highly functional nanomicelles are often assembled of complex building blocks, sophisticated multi-step processes are required for their synthesis. They entail a variety of intermediate polymers and the application of complex chemistry, which can influence not only the manufacturing process and production costs, but also the therapeutic efficacy (drug incorporation and micellar stability) and safety of the final nanoformulation. Therefore, a complex interplay of quality attributes influencing each stage of nanomicelle development should be considered and analysed to assess the overall risks, costs, and benefits, and Quality by Design approach is called for to optimize both design and manufacturing processes and ensure overall good manufacturing practice [18].

In this review, the elements for physicochemical characterization and preparation of drug loaded PM and their applications in cutaneous drug delivery, as well as the skin anatomy and drug delivery pathways when it comes to topical formulations, are described. 

## 2. Polymeric Micelles Physicochemical Characterization

**Figure 1:**
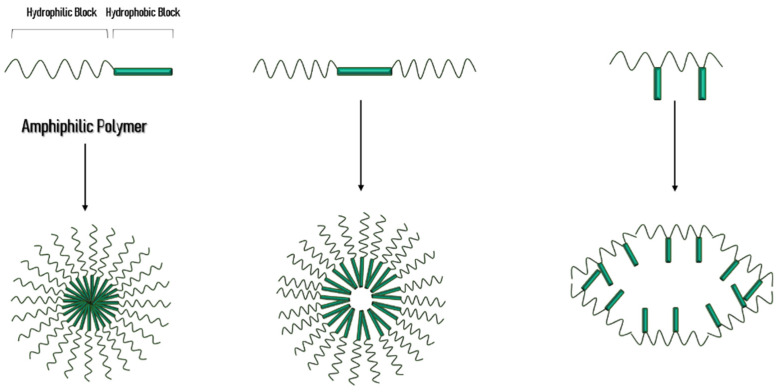
Schematic representation of the polymeric micelles formed by different polymers.

Polymeric micelles are nanoscale drug-delivery systems, normally between 10 and 100 nm, composed by amphiphilic block copolymers with hydrophilic (A) and hydrophobic (B) blocks self-assembled in aqueous solutions, creating a two-phase structure, commonly spherical, characterized by a hydrophobic inner core and a hydrophilic outer shell. Some of the applications of PMs are based in this core-shell architecture that provides protection to the hydrophobic portion, and, concomitantly, the therapeutic load, against biological invasion, while the hydrophilic shell also reduces protein adsorption on the micelle surface that can lead to systemic circulation clearance. As schematized in Figure 1, micelle-forming copolymers are typically constituted by di-block (A–B) or tri-block (A–B–A) copolymers, such as poloxamers and poloxamines, graft copolymers with different hydrophobicity (e.g., G-chitosan) or ionic copolymers (e.g., poly(ethylene glycol)-poly(ε-caprolactone)-g-polyethyleneimine). When prepared in organic solvent, under specific conditions, the copolymers can self-assemble into a reverse micelle, originating a hydrophilic inner core and a hydrophobic outer shell. The self-assembling of PM is a reversible process and it is dependent on the critical micelle concentration (CMC), described as the minimum concentration of polymers in solution leading to micelles formation. In aqueous solution, amphiphilic molecules work separately as surfactants, but as the concentration of copolymers increases, they start aggregating due to the saturation of the bulk solution, reaching CMC. Thus, micelles are stable for concentrations above CMC, but disassemble when the system experience dilution to values under CMC [8,9,12,13,19].

This parameter is one of the most important elements for PM characterization. The determination of CMC value in PM cannot be achieved by techniques such as interfacial tension, conductivity, and osmotic pressure because of the very low CMC values. Gel permeation chromatography is also limited in the determination of CMC due to adsorption of polymers on the column. Dynamic light scattering method is utilized to determine the hydrodynamic diameter of PM but in the quantification of CMC light-scattering technique is valuable but insufficient, since it can only determine CMC if the micellization occurs in a restricted concentration range, higher than the CMC value for block copolymers in water. However, CMC in PM can be determined by pyrene fluorescence, a highly sensitive technique, based on the portioning phenomenon of pyrene molecules [8,13].

Several properties of the copolymers can produce variations in CMC values. Wichit et al., for instance, studied the influence of the length of hydrophobic and hydrophilic fragments on CMC value, demonstrating that when the hydrophilic fragment was kept constant and the hydrophobic portion length was increased, that resulted in a lower CMC, explained by a higher number of interactions among the hydrophobic fragments, easily reaching a micellization equilibrium state. Lower CMC values are often related with an enhancing of the system stability, that can be retained even when diluted in the blood circulation system [13,20].

To quantify the success of PMs as nanocarriers in entrapping and delivering the active pharmaceutic ingredient (API), some calculations are of great importance, especially as a standardized measure when comparing different API-loading systems and delivery nanoformulations. When characterizing the PM-based nanocarrier systems, the loading capacity (Equation (1)), the incorporation efficiency (Equation (2)) and the delivery efficiency (Equation (3)) must be calculated according to the following equations [21,22,23].

(1) $$\mathrm{Loading}\;\mathrm{capacity}\;\left(\mathrm{LC}\right)\left(\%\right)=\frac{\mathrm{amount}\;\mathrm{of}\;\mathrm{API}\;\mathrm{incorporated}\;\mathrm{into}\;\mathrm{micelles}}{\mathrm{total}\;\mathrm{weight}\;\mathrm{of}\;\mathrm{micelles}}\times 100$$

(2) $$\mathrm{Incorporation}\;\mathrm{Efficiency}\;\left(\mathrm{IE}\right)\left(\%\right)=\frac{\mathrm{amount}\;\mathrm{of}\;\mathrm{API}\;\mathrm{incorporated}\;\mathrm{into}\;\mathrm{micelles}\;}{\mathrm{amount}\;\mathrm{of}\;\mathrm{API}\;\mathrm{introduced}}\times 100$$

(3) $$\mathrm{Delivery}\;\mathrm{Efficiency}\;\left(\mathrm{DE}\right)\left(\%\right)=\frac{\mathrm{amount}\;\mathrm{of}\;\mathrm{API}\;\mathrm{delivered}\;\mathrm{per}\;\mathrm{area}\;\mathrm{unit}}{\mathrm{amount}\;\mathrm{of}\;\mathrm{API}\;\mathrm{applied}\;\mathrm{per}\;\mathrm{area}\;\mathrm{unit}}\times 100$$

The size and diameter of PM, around 100 nm, is one of the most interesting parameters regarding these nanocarriers. Their size can be influenced by the length of both hydrophilic and hydrophobic blocks of amphiphilic copolymers, as well as by their molecular weight and the number of amphiphile aggregation number. Dynamic light scattering, atomic force microscopy, and transmission electron microscopy are the most commonly used techniques to measure the hydrodynamic size and polydispersity of micelles, while also characterizing their morphology [24,25]. 

Micelles usually have a spherical morphology. However, depending on the copolymers selected, the self-assembled system can origin a worm-like or cylindrical micelle morphology, polymer vesicles, or polymersomes. The structure of the formed PM is related to the hydrophilic–lipophilic balance (HLB) of the copolymers and the solvent selected for micelle preparation [26,27].

The zeta potential of micelles, with a common absolute value of 20–50 mV, is a parameter that contributes to the characterization and stability profile of the PM, as micelle surface charge affects the diffusion of particles when a micelle solution is placed under the effect of an electric field and it can also show influence the interactions with the biological environment, in endocytosis and exocytosis mechanisms [26,28,29]. 

### 2.1. Drug Loading and Micelle Preparation Methods

**Figure 2:**
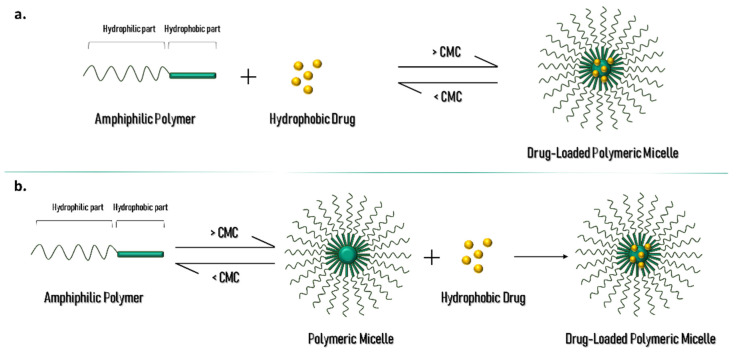
Schematic representation of PM and drug-loading process in (**a**) one step and (**b**) two steps.

The characteristics of PMs, described above, make them a promising candidate to be used as a nanocarrier to improve the cutaneous penetration of poorly soluble APIs or cosmetic ingredients. The solubility of API and the copolymer in water have great relevance in the selection of the PM preparation method. Some other properties need to be taken in consideration when developing PM, like the HLB, molecular weight and copolymer characteristics. Assessing the physicochemical profile of the substances is important to determine the preparation technique, where the API can be incorporated simultaneously with the micelle formation, or in a second step, as shown in Figure 2. In fact, the incorporation of API in the inner hydrophobic core of PM can occur by physical entrapment or chemical conjugation, depending on the method selected. However, the physical method is better succeeded in drug incorporation than the chemical method, that requires a specific functional group of the API to establish a covalent bond with the hydrophobic portion of the micelles, therefore causing its incorporation in the core [3,8,11]. 

#### 2.1.1. Direct Dissolution

Direct dissolution is the easiest technique to prepare PM, but dialysis, freeze-drying, oil-in-water emulsion, solid dispersion, and film hydration or solvent evaporation are also commonly used. In this simple preparation method, the amphiphilic copolymer and the drug, added in excess, are dissolved in an aqueous solvent. At or above CMC, the copolymer and drug self-assemble, creating a loaded PM. This technique presupposes that both the drug and the copolymer are water-soluble and it is associated with a low drug loading, although increasing the temperature of the system might reduce this disadvantage [1,9]. 

#### 2.1.2. Dialysis

If the selected copolymers have low water solubility, the dissolution of copolymer and drug can be executed in a water-miscible organic solvent, that posteriorly is replaced with deionized water through a semipermeable membrane, inducing micelle formation. The solvent removal is a slow process that often requires up to 36 hours, and the IE and size of the particles are dependent of this phase [1,9,30].

#### 2.1.3. Freeze-Drying

Tert-butanol or other freeze-dryable organic solvents are used in the freeze-drying method. The copolymer and the drug are dissolved in a water/tert–butanol mixture, that suffers freeze-drying process to remove the solvents used. Next, water is added to the resultant powder and the micelle formation occurs. The final product can, however, have a residual presence of the organic solvents used in the process, constituting a disadvan tage this preparation method [1,9].

#### 2.1.4. Oil-in-Water Emulsion

The main challenge in oil-in-water emulsion method is to totally remove the free drug and the organic solvent. In this micelle preparation technique, a water-immiscible volatile organic solvent, such as chloroform, is added slowly to the aqueous medium, forming an emulsion. This aqueous medium contains the copolymer and drug, and the micelle formation happens after agitation and solvent evaporation [1,9,30]. 

#### 2.1.5. Solid Dispersion

A volatile solvent is selected to dissolve both the copolymer and the drug. After the evaporation of the solvent, a polymeric drug containing matrix is formed. Subsequently, the matrix is heated and hot water is added, leading to micelle formation. This method is also designated as solution casting [31]. 

#### 2.1.6. Solvent Evaporation

**Figure 3:**
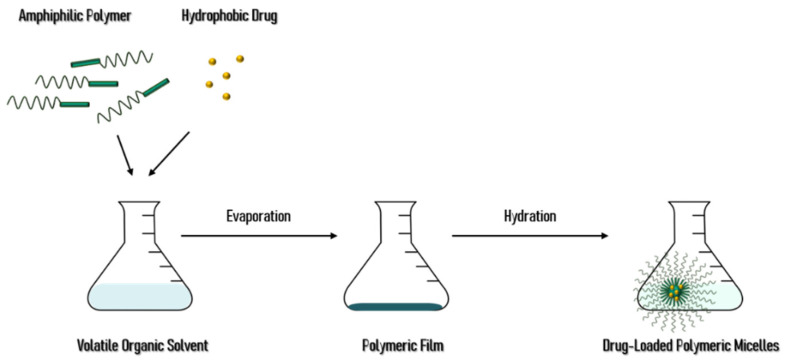
Schematic representation of the solvent evaporation process for polymeric micelle’s preparation.

Also known as film hydration method, solvent evaporation process demonstrates a high incorporation efficiency in copolymers with less water solubility, being only selected for copolymers with low HLB values. This method, represented in Figure 3, consists in dissolving the drug and copolymers in a volatile organic solvent or a mixture, that is afterwards evaporated, forming a thin polymeric film at the recipient’s bottom. The film is then hydrated with water by sonication or stirring [1,9,30].

#### 2.1.7. Co-Solvent Evaporation

This method of polymeric micelle preparation combines the solvent evaporation followed by dialysis, possibly overcoming some of the disadvantages of separate preparation techniques. The selection of the solvents in this method, the organic to aqueous ratio or the order of phase addition, can manipulate the self-assembly conditions, improving the IE and average size of the PM prepared. For instance, in the co-solvent evaporation method, Aliabadi et al. confirmed that the addition of acetone to water in a low organic to water phase ratio was able to increase the IE and conduct to smaller average diameter PM [32].

Still, a theoretical equilibration of the drug and micelles in an aqueous medium does not always translates in high levels of incorporated drugs, as described above, and polymeric micelles, despite of having a large drug entrapment capacity, cannot always load all the drug available. Lapteva et al. demonstrated that when the concentration drug was increased, micelle formulations could exhibit a higher loading capacity. However, due to the supersaturation of the drug in the micelle hydrophobic core during the micelle formation process, the incorporation efficiency of micelles decreased, and drug precipitated [14,18]. 

**Figure 4:**
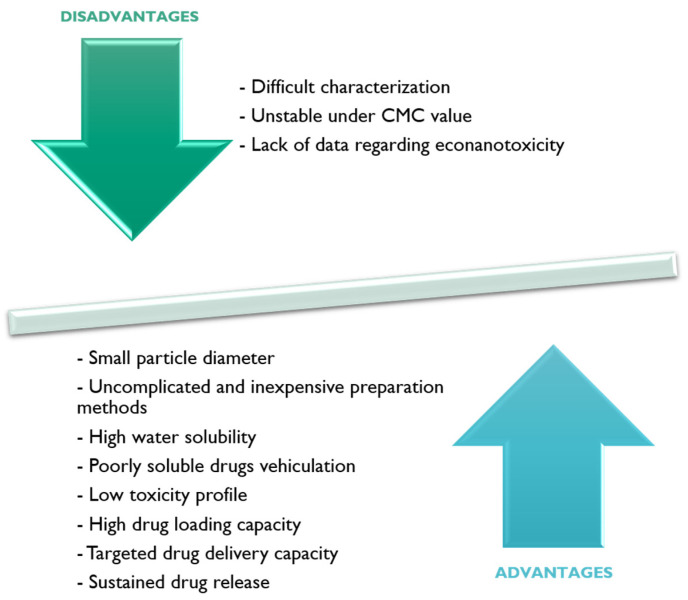
Summarized schematization of the principal advantages and disadvantages for PM-based carrier systems.

Of all the advantages and disadvantages described for PM-based carrier systems, the main ones are summarized in Figure 4.

## 3. Skin Anatomy and Physiology

**Figure 5:**
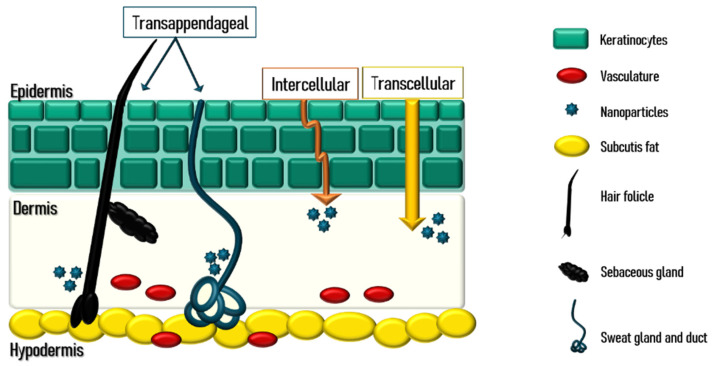
Schematic view of skin layers and drug-delivery routes.

Human skin is a multilayer soft tissue that covers almost all of the body surface, taking up to 16% of the body weight and a surface area of 1.8 m^2^. It is an integument and has protective functions against xenobiotic compounds and external aggressions, such as ultraviolet (UV) radiation, pressure, stress, or trauma. It also receives sensorial stimulations from the environment, provides thermoregulation of the body, and prevents transepidermal water loss (TEWL) [33]. The skin thickness can variate according to the age and sex of the individuals [34]. Also, the human skin has topographic differences regarding its thickness, that varies depending on the anatomical site of the body, and its hair distribution distinguishes human skin from the skin of all the other land mammals [34]. While in some areas, the hair is apparently vestigial, in some other areas of the body, the epigamic areas, hair grows abundantly, in a phenomenon linked with social and sexual communication. The skin is composed of three layers: epidermis, dermis, and hypodermis, as schematized in Figure 5.

Epidermis is the outer skin layer with a thickness variating from 0.05 mm in the thinner areas, such as the eyelids, to 0.8 ± 1.5 mm on the palms of the hands and soles of the feet. It is a stratified epithelium that acts as a physicochemical barrier between the exterior environment and the interior of the body, constituted majorly by: keratinocytes, that produce protein keratins; desmosomes, with ligand properties that connect keratinocytes together; melanocytes, productors of melanin, the pigmentation compound in hair and skin; and Langerhans cells, responsible for the immune response against pathogenic agents. Keratinocytes have several stages of maturation, originating four different layers in the epidermis, from the deeper and in the direction of the surface: the germinativum cell layer (*Stratum basale*), the spinous layer (*Stratum spinosum*), the granular cell layer (*Stratum granulosum*), and the *Stratum Corneum* (SC), the outermost layer of the epidermis that mainly contains lipid components like ceramides, cholesterol, and fatty acids, contributing to a water-preserved environment in the skin [35,36]. 

The corneocytes in SC are distributed in a structure often designated as a ‘bricks and mortar’ arrangement, due to the cells being flattened and anuclear, densely packed within the extracellular lipid matrix, and disposed in layers. The epidermis does not have blood vessels present, and its nutrition is only provided by diffusion from the dermoepidermal junctions, that separate the epidermis basal layer (*Stratum basale*) and the dermis layers [37]. 

The dermis is the skin middle layer and the supportive cell matrix for the skin, made up of fibroblasts that produce collagen fibres and glycosaminoglycans, two macromolecule components of the skin extracellular matrix. Collagen maintains the turgidity of the skin due to its water-holding capacity, creating a network of extendable fibres responsible for the elasticity and flexibility properties of the skin. The dermis, especially the deeper layers, is rich in blood vessels, emerging from the hypodermis layer [35].

The hypodermis of the skin, also known as the subcutis, is constituted by loose connective tissue and fat. It is also in the hypodermis layer that Meissner’s and Pacinian corpuscles are located, responsible for the reception of the touch and vibration stimulus, respectively [35]. 

Apart from the skin layers, Pilosebaceous Units (PSU), nails, and sweat glands are, as well, constituents of the human skin that are of great importance in the topical drug delivery, as they can be seen as entrances to substitute the penetration through the skin cells. PSU includes the hair follicles and the sebaceous glands, which secret sebum to lubricate hair and skin. Nails occur on the tips of the fingers with a protection purpose and they are dense plates of hardened keratin. Sweat glands have a thermoregulation function and can also excrete body wastes and products resulting from metabolism and are extended from the dermis to subcutaneous [35]. 

### Skin Routes for Drug Delivery

During skin penetration, drug molecules can experience several fates, depending on their properties. While some compounds are not able to deeply penetrate the skin, and suffer deposition in the surface layers, some other substances and carriers permeate to deeper stratus, with the possibility to attain the adipose tissue and muscles and, in some cases, reach the systemic absorption. The physicochemical profile of the API and carriers, such as particle size, surface charge, lipophilicity, solubility, and fluidity, translates in different penetration rates through existing pathways [2]. The skin penetration is performed simultaneously by different routes, but, due to its particularities, there is always a main transportation path. There are three pathways for drugs to penetrate the skin: *transcellular, intercellular,* and *transappendageal* [3]. 

In the transepidermal route, the intercellular pathway is described as the main transport pathway of most drugs, best suited for uncharged lipophilic molecules and, thus, the most likely to occur. Substances diffuse through the lipid matrix between the keratinocytes, in a pathway modulated by the convoluted structure that corneocytes can form. Large molecules get physically restricted within the lipids and are not able to penetrate, while small molecules traverse the alternating lipids and aqueous mediums, and diffuse through [35,37,38]. 

Transcellular route occurs when penetrant compounds diffuse through the corneocytes of the SC. However, there are not numerous drugs capable of penetration via the transcellular route, as the penetrant needs to pass through the phospholipid bilayer of each cell and its cytoplasm, resulting in several partitioning and diffusion steps. This drug pathway is more important as a polar route or when the drug is formulated with a penetration enhancer able to change the keratin structure and, consequently, to increase the corneocytes permeability [3,38]. 

Transappendageal route, also known as the shunt route, occurs when a drug utilizes the appendageal elements of the skin to penetrate, such as the sweat glands, PSU, and pores. It was described as the pathway with the less significant contribution to the skin penetration, as the appendages only cover 0.1% the skin area. Despite of that fact, this is an important pathway of penetration for drugs with low permeation in the transcellular and the intercellular routes. Other opportunity regarding the shunt route is the targeted drug delivery, specifically for PSU and sweat glands affecting disorders. The application of targeted drug delivery in hair follicles and sebaceous glands can also improve the clinical efficacy of several topical treatments, as a smaller amount of drug is needed, reducing the potential local and systemic adverse effects. According to that, formulating drug carriers with targeted API delivery can improve the API’s safety profile, enhancing the patient compliance to the treatment as well [3,38]. 

Thus, when formulating pharmaceuticals and cosmetic products for skin application, the main challenge is to overcome the physicochemical barrier properties of the skin cells and layers, enhancing penetration in transdermal pathways or targeting the drug delivery to specific skin organelles, such as hair follicles [3,35]. 

## 4. Applications of Polymeric Micelles in Cosmetic and Topical Drug Formulations

In the past years, several studies regarding the application of PM in topical pharmaceutical and cosmetic products have been conducted, with the most representative examples presented in this chapter, and summarized in Table 1.

### 4.1. Anti-Ageing

Over time, the entire human body experiences the inexorable intrinsic ageing process, and skin is not an exception. The increase of life expectancy around the globe elevates the interest around the skin ageing process. Though this process is not fully understood, it appears to be caused by oxidative stress, genetic and mitochondrial DNA mutations, decrease of multiple hormone levels, and shortening of telomeres. In addition, UV-radiation plays an important role in the ageing process of the portions of skin directly exposed to sunlight. Skin ageing can also be regulated by exogenous factors and the environment, such as tobacco smoke, airborne particulate matter, malnutrition, infrared and UV-radiation, as mentioned. Oxidative cell metabolism and formation of reactive oxygen species (ROS) and free radicals can be an important element in the endogenous and photo-induced skin ageing, leading to the transcription factor c-Jun mitogen-activated protein kinases, promotion of matrix metalloproteinases (MMP) expression and inhibition of procollagen-1 expression, which contributes to the deposition of elevated levels of partially degraded collagen. The epidermal turnover also has age-related changes and corneocytes turnover decreases. As time goes by, the collective of all the elements, endogenous and exogenous, preventable and unpreventable, leads to skin dryness, wrinkling, epidermal atrophy, decreased collagen levels, and loss of elasticity [40,49,50,51]. 

#### 4.1.1. Oleanolic Acid

Oleanolic Acid (OA) is a pentacyclic triterpenoid isolated from plants, such as *Olea Europaea*, *Eugenia jambos,* and *Gentiana lutea*. As an active substance in anti-ageing formulations, it promotes the synthesis of ceramides, pro-collagen, and filaggrin. It also inhibits the activity of MMP-1, an important enzyme in the collagen degradation process. Due to its low aqueous solubility and permeation in the skin, OA is poorly absorbed and, therefore, and PMs have been studied as an alternative for the colloidal drug carriers used as permeation enhancers [39]. 

An et al. incorporated OA in PM in order to verify if nanoencapsulated OA was more efficient in alleviating wrinkles in human periocular skin. OA was dissolved in solubilizer Capryol^®^ 90 (propylene glycol caprylate), chosen after a solubility test conducted with several solvents. PMs loaded with oleanolic acid (PMO) were prepared with the formulation compositions G (0.05 *w*/*w*% OA; 2 *w*/*w*% Capryol^®^ 90; 6 *w*/*w*% Poloxamer 407) and H (0.05 *w*/*w*% OA; 2 *w*/*w*% Capryol^®^ 90; and 7 *w*/*w*% Poloxamer 407), as described in Table 2 [39].

By showing a higher encapsulation efficacy and a lower average particle size, PMO-H was selected as the main active ingredient of the cosmetic formulation in a clinical trial with 23 female subjects, aged between 30 and 65 years old, and wrinkles in the periocular area. They applied the sample on one side of the face and the control on the other side, daily, during the eight-week trial period. The subjects were evaluated every four weeks, where specialists concluded that nanoencapsulated OA proves to be more effective in alleviating skin wrinkles than nonencapsulated OA, with no skin irritation registered [39]. 

#### 4.1.2. Coenzyme Q10

Hyaluronan (HA) is a linear non-sulphated polysaccharide glycosaminoglycan studied for almost a century, and is one of the major components of the extracellular matrix in vertebrate connective tissues, found in abundancy in the brain, synovial fluid, cartilage, and skin which is the main reservoir of HA [52]. Present in both the epidermis and dermis, HA in skin is approximately one third of the total amount of HA expected to be found in the human body [40]. HA levels, as well as skin sebum production levels, can decrease with age, resulting in imbalance of the hydrolipidic mantle, which can disrupt SC and accelerate the skin ageing [53]. 

Due to its relevance in human body, a biodegradable and biocompatible low-molecular-weight HA has been studied as a drug-carrier since it acts as skin penetration enhancer and induces cell uptake by receptor-mediated endocytosis [6]. When grafted with fatty acids, HA can penetrate the SC and reach deeper layers of skin by transcellular route, and can interact with cellular membranes by both active and passive uptake mechanisms. 

Tha ability of HA to deliver APIs into different layers of skin was evaluated in different model systems. Šmejkalová et al. studied the delivery capacity of Nile Red (NR) incorporated in HA modified by cis-oleic acid (oleyl-hyaluronan HAC18:1) and hexanoic anhydride (hexyl-hyaluronan HAC6) nanomicelles [40]. NR, a hydrophobic substance selected for its great fluorescent traceability, was incorporated into micelles by solvent exchange method. The delivery efficacy of NR-loaded HAC6 and HAC18:1 PMs was compared with the mixture of NR and HAC6 or HAC18:1 emulsions in oleic acid oil, as well as the NR in oleic acid oil and NR dispersed in saline phosphate buffer. Even though the loading capacity for NR was low, it served the purpose of the study due to its strong fluorescent signal and the ability to track PM penetration. The results have shown that the NR-loaded micelles were able to reach deeper layers of the epidermis and dermis in samples of porcine skin, when compared to the other tested formulations [40]. HAC18:1 micelles loaded with FRET pair dyes were used to investigate the fate of micelles as they penetrate into deeper skin layers. FRET effect was found to decrease with skin depth as a result of increased distance between core-loaded DiI (fluorescent lipophilic acceptor probe) and DiO (fluorescent lipophilic donor probe), indicating progressive micelle disruption. 

In another model system, micelles composed of HAC18:1 or HAC6 covalently labelled with Nile Blue (NB) and loaded with the model hydrophobic compound curcumin were used to investigate the mode of internalization od HA-based micelles by fibroblasts and keratinocytes [6]. The accumulation and uptake mechanisms were strongly dictated by different physiologies of tested cells. Additionally, HAC5 micelles prefer more active intracellular transport than HAC18:1 micellar system. Although the internalization mechanisms, among others, involve clathrin-mediated endocytosis, the influence of CD-44 receptor of HA was not confirmed, suggesting involvement of other receptors or fully unspecific active uptake. 

PM-based delivery systems could, therefore, be useful vehicles for delivery of hydrophobic cosmetic or pharmaceutical compounds. 

The antioxidant effect of Coenzyme Q10 (CoQ10), a highly lipophilic non-enzymatic substance with limited topical bioavailability, has been studied in anti-ageing formulations [51]. Endogenous levels of this coenzyme decline after the age of 30 and, therefore, developing formulations that can deliver exogenous CoQ10 to the epidermis, despite its high molecular weight and low aqueous solubility, have a great potential in anti-ageing products [50]. 

Šmejkalová et al. prepared CoQ10 loaded PMs by solvent evaporation method, adding 6 mg of CoQ10 dissolved in chloroform to 1% HAC6 or 13 mg of CoQ10 to 1% HAC18:1, in aqueous solution [41]. Organic solvent was removed by rotary evaporation, loaded particles were filtrated, and the loaded PM filtrate was freeze-dried. Supercritical fluid chromatography with UV detection was chosen for quantification of the amount of incorporated drug and the loading capacity was calculated according to Equation (1). In human fibroblasts, in vitro assessment to test the protective anti-oxidative activity of CoQ10 after treating the skin with hydrogen peroxide concluded that only CoQ10 loaded in HAC18:1 could protect the skin against oxidative stress. Unloaded HAC18:1 and free CoQ10 in the same concentrations were not able to exhibit the same result and could not prevent the negative change in the mitochondrial potential. Afterwards, CoQ10-loaded HAC18:1 micelles were formulated in a o/w cream for a four-week in vivo trial with human volunteers divided in four groups: control group, unloaded HAC18:1 group, free CoQ10 group, and CoaQ10 loaded HAC18:1. The subjects applied the cream once a day and their skin’s hydration, elasticity, and trans epidermal water loss (TEWL) were tested after each week using MPA 580 equipped with several probes. Researchers did not find significant differences in the elasticity and TEWL parameters between groups, but in CoQ10 loaded HAC18:1 PM group skin hydration was 2–3 times higher compared to others, revealing the potential of PM as an improver of the bioactivity of the payload [40]. 

### 4.2. Acne Vulgaris

*Acne vulgaris*, a disease that occurs in the pilosebaceous unit (PSU), is estimated as being the eighth most prevalent disease worldwide, involving over 80% of Western European adolescents and 9.4% of the global population, affecting not only the skin and physical appearance, but also the patient’s self-esteem, resulting sometimes in psychological complications [41,54,55]. Patients with acne experience an increased sebum production, an abnormal proliferation and differentiation of keratinocytes, a hyperproliferation of *Propionibacterium acnes*, and an inflammatory response that is initiated by bacterial antigens and cytokines [56]. These alterations can weaken the skin barrier function and result in an intensification in TEWL level and a decrease in skin hydration. Guidelines for pharmacotherapeutic treatment of *acne vulgaris* include topical and oral options, reserving the last one for more severe cases, due to the systemic adverse effects that often occur. Considering the etiology of acne, the API in topical application formulations must reach the follicular epithelium to exert its therapeutic effect, making it an ideal candidate for targeted follicular delivery. When targeted drug delivery to the PSU is not achieved, treatment lacks efficacy and increases the risk of local adverse effects [38,41]. 

#### 4.2.1. All-Trans Retinoic Acid

All-*trans* retinoic acid, also known as tretinoin (TRN), is one of the most widely used retinoids for acne topical treatment, that can regulate epithelial cells growth and differentiation and proved to be effective as a treatment in several other dermatological pathologies such as psoriasis and photo-aging. Patients treated with TRN often suffer from undesirable local side-effects described as retinoid dermatitis, with symptoms variating from irritation, erythema, and itching to burning and desquamation. TRN is highly lipophilic and suffers degradation by heat, light, and isomerized agents, leading to the formation of isomers or oxidation products with lower bioactivity in the formulated products. Of the different photoisomers, the only substances that might be active and possess retinoid-like pharmaceutical and pharmacological effects are 13-*cis* retinoic acid, isotretinoin, and 9-*cis* retinoic acid, alitretinoin. When oxidized, TRN forms 4-hydroxy-, 4-oxo-, and 5,6-epoxy retinoic acids, all showing reduction of effectiveness when compared with TRN [20,41]. 

Wichit et al. conducted a study attempting to protect degradation of TRN from oxidation, utilizing PMs composed of poly(ethylene glycol)-conjugated phosphatidylethanolamine (PEG-PE), as poly(ethylene glycol) (PEG) and phosphatidylethanolamine (PE) are proven to enhance skin drug permeation and, possibly, reduce surface tension of the SC. Several PEGs with different mass weights (750 Da and 5000 Da) were conjugated with a series of diacyl chains of PE, studying the influence of the hydrophilic and hydrophobic copolymer blocks on CMC, size of PM and loading capacity. It was found that the increase of the hydrophobic fragment while maintaining the hydrophilic block’s length, reduces CMC value. As to micelle size, longer hydrophilic blocks (PEG_5000_) origin PMs with an average of 17 nm, while PM formed with shorter hydrophilic blocks (PEG_750_) were able to reach diameters of less than 10 nm.

**Figure 6:**
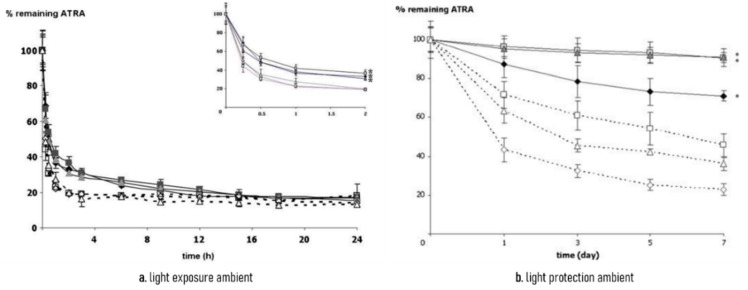
Remaining ATRA (%) in loaded PEG_750_-DPPE micelles (closed symbols) and 75% methanol/HBS solution (open symbols). Diamonds represent oxygen-filled environments, squares represent nitrogen-filled environments, and triangles represent ambient air (adapted from [20]). (**a**) light exposure ambient; (**b**) light protection ambient.

Oxidation protection of TRN was demonstrated with PEG-PE micelles produced from 750 Da PEG conjugated with dipalmitoyl phosphatidylethanolamine (PEG_750_-DPPE), the formulation with the highest loading capacity, and was compared to TRN in 75% meth anol/HBS solution used as a control. The samples were placed in containers filled with oxygen, nitrogen, or ambient air, at room temperature, with or without light exposure. The remaining percentage of TRN, that did not suffer oxidation, was quantified by high performance liquid chromatography using the aliquots collected at given times during the experiment. It was established that PEG_750_-DPPE micelles were able to slow the degradation of TRN in the presence of atmospheric oxygen, as demonstrated in Figure 6 [20].

Lapteva et al. experimented PSU-targeted delivery of TRN loaded in PM, using a biodegradable and biocompatible copolymer—diblock methoxy-poly(ethylene glycol)-poly(hexyl-substituted lactic acid) (MPEG-dihexPLA)—that after degradation forms 2-hydroxyoctanoic acid and lactic acid, both non-toxic substances [42]. In this experiment, TRN was quantified along with its photoisomers, in a collective term below designated as TRNi, and the efficiency on both cutaneous and targeted PSU delivery of TRNi from TRN loaded MPEG-dihexPLA were compared with marketed formulations Retin-A Micro^®^ (0.04%) and Effederm^®^ (0.05%). The PM were prepared by solvent evaporation in three formulations—A, B, C—with different TRN loading—20, 25, 30 mg_TRNi_/g_copolymer_. Formulations B and C were able to incorporate a higher TRNi content, but formulation A was selected for use in studies due to its better stability. The micelles presented diameters below 20 nm and were not affected in size when Quinoline Yellow (QY) was added as a photostabilizer. QY was selected to provide photostabilization of TRN, due to its maximum absorption wavelength (420 nm) which is coincident with the wavelength that triggers photoisomerization of tretinoin. Samples in experiment were applied on porcine ears and human skin collected during breast reduction in a male subject, containing terminal hairs. In full-thickness skins, both porcine and human, PM formulation had a significantly higher DE than Retin-A^®^ Micro and a similar DE to Efferderm^®^ solution; when concluding about PSU targeted delivery, TRN-loaded MPEG-dihexPLA efficiency was comparable to Retin-A^®^ Micro and colloidal formulations, although it was inferior that Efferderm^®^. Nanoparticulate formulation in study was, however, more efficient than the microspheres, demonstrating the importance of the size of particles in cutaneous drug delivery and indicating the potential of PM as nanocarriers for TRN, leading to improvements in *Acne vulgaris* treatments and better skin irritation profile. 

#### 4.2.2. Adapalene

Adapalene (ADA) is a lipophilic molecule and a third-generation retinoid frequently used in the topical treatment of mild to moderate *Acne vulgaris*. Unlike retinoic acid and other retinoids, ADA is photochemically stable, and better tolerated when compared to first-generation retinoids. However, patients treated by topical formulations containing ADA can still experience some adverse effects including local dryness, erythema and itching, sometimes affecting patient compliance. Given the high lipophilicity and low aqueous solubility of retinoids, the development of effective and pleasing formulations can be a challenge [42,56].

Kandekar et al. used PMs to study the selective delivery of ADA to the human hair follicle under finite dose, in order to arrange an alternative formulation with improved efficacy and reduced adverse side effects. The use of finite dose conditions in in vitro studies tends to mimic more accurately “real” conditions, as infinite dose conditions risk overestimating the amount of drug delivered in vivo. The use of smaller amounts of formulation in finite dose conditions also decreases the effect of excipients, being more representative of in vivo behaviour. The ADA entrapted in D-α-tocopheryl polyethylene glycol 1000 succinate (TPGS) PMs were prepared using the solvent evaporation method, applying acetone as the solvent. 

**Figure 7:**
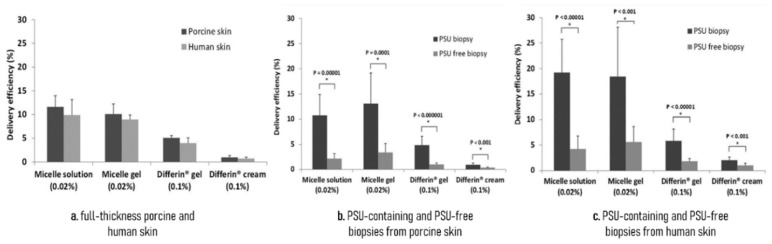
Comparison of ADA delivery efficiency of formulations (12 h after application) in finite dose of the ADA formulations, corresponding to 2 and 10 µg/cm^2^ of ADA for the micelle and marketed formulations, respectively. Reprinted with permission from [42]). Copyright Royal Society of Chemistry 2018.

ADA-TPGS (<20 nm) were developed into a stable micelle solution and jellified in sodium carboxymethyl cellulose (NaCMC) to improve skin adhesion, accurate dosing, rheological properties, and to facilitate application. The micelle solution (0.02% ADA) and the micelle gel (0.02% ADA) were tested against marketed formulations, Differin^®^ gel (0.1% ADA) and Differin^®^ cream (0.1% ADA), to compare ADA skin delivery and ADA targeted PSU delivery in porcine and human skin. The DE was calculated according to the Equation (3) and results shown that ADA-TPGS is capable of PSU-targeted delivery, at a lower ADA content versus marketed formulations (Figure 7), which might provide less skin irritation and adverse effects, improving patient compliance. This study also verifies that TPGS-based micelles can serve as nanocarriers for poorly soluble drugs in formulations aimed at treatment of PSU related pathologies. Nevertheless, those promising results need further confirmation in clinical trial [42,57]. 

#### 4.2.3. Benzoyl Peroxide

Since the 1960s, and due to its multifactorial action, benzoyl peroxide (BPO) has been used for the topical treatment of mild to moderate acne cases, available in gel formulations as a non-prescription medication, prescribed in monotherapeutical scheme or combined with other APIs. BPO is a lipophilic organic peroxide with low aqueous solubility that has keratolytic, mildly anti-inflammatory, and antibacterial properties, the last one owing to the formation of ROS when the BPO decomposition occurs and their posterior reaction with bacterial proteins. Patient compliance is sometimes low, and retinoid-like adverse reactions are frequent [43,55]. 

Kahraman et al. developed micellar nanocarriers of BPO with Pluronic^®^ F127, a non-ionic polymer approved by the Food and Drug Administration (FDA), prepared by thin film hydration method [43]. They were characterized prior the in vitro study, and the formulation of PM with the highest encapsulation efficiency were accomplished from a 1.0–0.015 (*w/w*) ratio of Pluronic^®^ F127 and BPO, characterized by an average size of 25 nm and a narrow polydispersity index, with a relatively low irritant potential that was estimated with the cytotoxicity essay. In this study, the deposition of BPO from PM was compared to Benzamycin^®^, a marketed gel formulation, applied to dorsal porcine skin, that was selected for its resemblance with human skin in hair follicle structure and SC thickness. The data collected demonstrate that Pluronic^®^ F127 was able to safely and efficiently deliver the drug by targeting the PSU, constituting a potential alternative as a drug carrier in BPO formulation, needing, although, further in vivo trials to support this in vitro and ex vivo conclusions. 

### 4.3. Psoriasis

Psoriasis is a chronic inflammatory autoimmune disorder, manifested by immunological and biochemical alterations that activate immune cells. Patients with psoriasis experience higher TEWL, an abnormal proliferation and disrupted differentiation of keratinocytes, with inflammatory lesions, linked with augmented levels of growth factors, chemokines, pro-inflammatory markers, and cytokines like interleukin 17 (IL-17), interleukin 23 (IL-23) and tumor necrosis factor α (TNF-α). It is not a life-threatening disease, and it does not affect the overall health of the patient, but it can interfere in quality of life and social interactions, resulting in some physical and psychosomatic disorders. Red or pink patches on the skin surface covered by silver or white scales are characteristic of plaque psoriasis. Physicians often recur to anti-inflammatory drugs for long-term administration, selecting topical over oral drugs in mild to moderate cases of psoriasis. Nevertheless, none of the available treatments can cure this disease completely with effectiveness and safety [1,21,23]. 

#### 4.3.1. Tacrolimus

Isolated from *Streptomyces tsukubaensis*, tacrolimus (TAC) is a macrolide immunosuppressant with a 10-fold greater in vivo immunosuppressive activity than ciclosporin A. TAC mechanism of action is similar to that of ciclosporin A, binding the immunophilin FKBP12 inside the activated T-cells, indirectly inhibiting the expression of interleukin 2 and immune response by several cytokines and T-cells. Systemic treatments with TAC proved to be effective in psoriasis cases, but they also have adverse effects. Topical applications of TAC, however, lack the demonstrated ability of TAC deposition in psoriasis plaques, being only possible under occlusion or in body areas with thinner skin, as that element eases the permeation. Some carrier systems that have been investigated to promote the bioavailability of TAC also possess increased transdermal permeation, therefore increasing the potential risk of systems side effects in vivo [21]. 

Lapteva et al. prepared MPEG-dihexPLA micelles using the solvent evaporation method, to serve as nanocarriers for TAC [21]. Different TAC concentrations were loaded to MPEG-dihexPLA (50, 100, 150, 200, 300, and 500 mg_TAC_/g_copolymer_) and characterized using Equations (1)–(3) and also evaluating their stability. Once more, the increasing of drug loading resulted in API precipitation out of the micelle.

**Figure 8:**
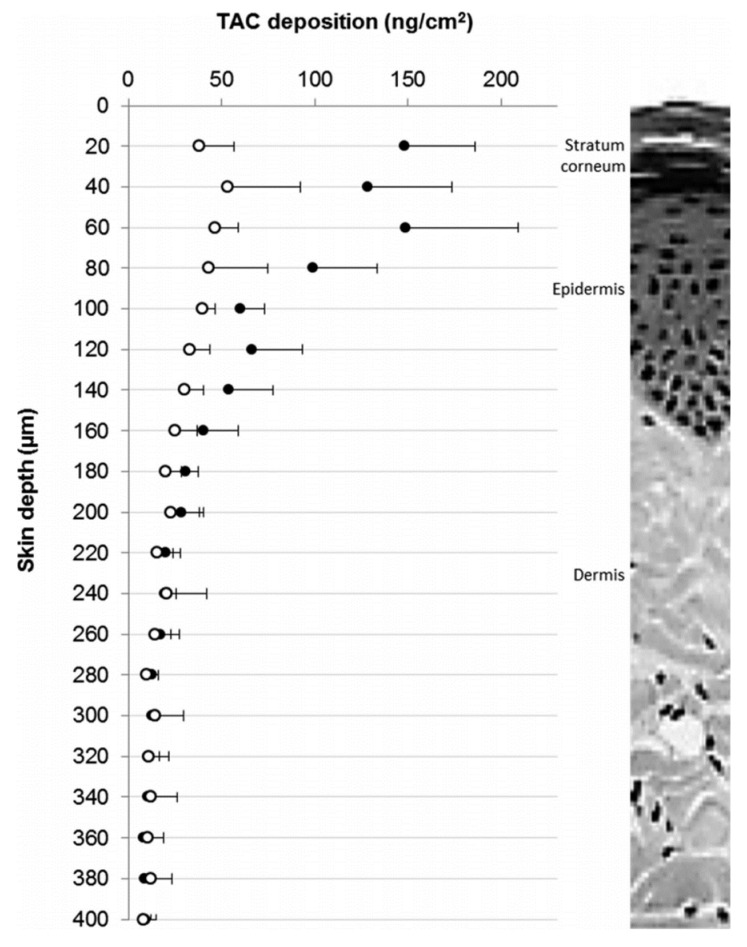
Cutaneous distribution profile of TAC in the upper layers of human skin (total depth of 400 µm and a resolution of 20 µm) after a 12 h application of the 0.1% micelle formulation (close dots) and Protopic (0.1% *w*/*w*) (open dots). Reprinted with permission from [21]. Copyright American Chemical Society 2014.

To compare the cutaneous bioavailability of TAC and its distribution profile along the skin layers, a stable formulation of 20 nm PMs containing 0.1% TAC, was compared with the one of the marketed formulations, Protopic^®^ (0.1% *w/w*), and applied to porcine ears. TAC deposition from the micelle formulation after 12 h was significantly superior (Figure 8), especially in the SC, viable epidermis and superficial dermis, with similar deposition in the deeper layers as Protopic^®^, suggesting that TAC loaded MPEG-dihexPLA micelles can increase clinical efficacy in psoriasis topical treatment without increasing the risk of potential systemic adverse effects. 

#### 4.3.2. Resveratrol

3,5,4’-trihydroxy-*trans*-stillbene, resveratrol (RES), is a polyphenolic phytoalexin compound and can be isolated from red wine, berries, and grape skin, with a studied anti-inflammatory, antioxidant, anti-proliferative immunomodulator, and anticancer activities. Its mechanism of action involves the activation of SIRT-1, a Sirtuin gene present in the skin, which modulates immune cells activity, decreases proliferation and promotes differentiation. It is a safer API for treatment of plaque psoriasis, that can overcome some of the existing concerns related to more traditional approaches. However, RES is categorized as class II of the Biopharmaceutics Classification Systems for its poor water solubility, that hamper RES bioavailability and topical delivery [23,58]. 

**Figure 9:**
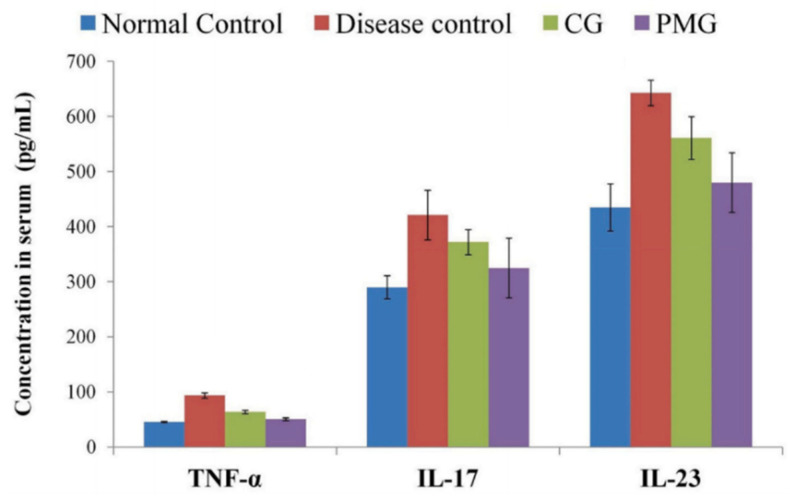
Serum cytokines concentration (TNF-α, IL-17, and IL-23) of different groups. Reprinted with permission from [23]. Copyright Elsevier 2020.

Khurana et al. [23] successfully conducted a study applying Quality by Design development to optimize RES-loaded PMs as an alternative therapy for the treatment of psoriasis plaques. RES-loaded PMs were prepared using Poloxamer F127 and P123 as copolymers, and film hydration as the selected preparation method. RES-PMs were posteriorly formulated in a PM carbomer-based hydrogel (PMG), for comparison with conventional hydrogel (CG)—distilled water instead of the micellar dispersion and the equivalent quantity of RES (10 mg). In vitro drug-release testing from PMG, CG, RES-PM, and RES solution demonstrated that CG and RES solution showed a burst release in the first 2 h, while RES-PM and PMG demonstrated a sustained release of up to 12 h, due to the slow diffusion of RES from the micelles. When comparing RES-PM and PMG for RES skin deposition, results showed that PMG achieved better skin permeation and RES deposition in deeper skin layers. In vivo trial with Swiss Albino mice, divided into four groups (control, disease, diseased animals treated with CG, and PMG, respectively) was also performed. After inducing psoriasis in the back of the mice with imiquimod for seven days and treating them for other week, their serum cytokine concentrations were measured, revealing that CG and PMG group had a decrease in the levels of IL-17, IL-23, and TNF-α, as described in Figure 9. However, PMG group showed a larger decrease in cytokines levels, as well as a spleen weight much more comparable in the control, psoriasis-free, group [23].

#### 4.3.3. Silibinin

Silibinin (SL) is a flavonolignan found in milk thistle, *Silybum marianum*, and also a silymarin component and possesses hepatoprotective action, along with antioxidant and antineoplastic activity, and can act as a phosphorylation signal inhibitor [49]. As psoriasis is often related with the activation of phosphorylation signals in keratocytes, SL efficacy as an alternative to the conventional treatment was studied by Chavoshy et al. [44]. A series of SL-loaded PM were prepared, and permeation through skin in psoriatic and non-psoriatic mice was evaluated. Similar to 4.3 posteriorly, mice with induced psoriasis were treated with SL the PM-based formulation and compared with the control group. Results showed that SL-PM treated mice experienced a reduction of the psoriasis area index by more than 78% as well as increased drug localization in the psoriatic plaques. Therefore, SL has proven to be a great candidate for alternative psoriasis treatment, especially formulated as a polymeric micelle nanocarrier.

### 4.4. Fungal Infections

Mycoses and other fungal infections distress more than 25% of population around the globe, affecting particularly immune-compromised patients, due to their age, clinical condition, pathologies, and concomitant bacterial, viral, and fungal infections. These opportunistic mycoses, often caused by *Candida spp., Cryptococcus neoformans, Pneumocystis jirovecii, Aspergillus spp.,* and *Trychophyton spp.,* are responsible for around 1,500,000 deaths every year. Fungal infection treatments include topical or/and oral administration of antifungal agents. Although oral therapeutic options are very effective, they can cause several toxic adverse effects and augment the risk of potential drug interactions [46,59]. 

#### Azoles’ Antifungals

Azoles’, a pharmacotherapeutic group of substances with antifungal properties, such as terconazole (TCZ), clotrimazole (CLZ), econazole nitrate (ECZ), and fluconazole (FLZ), are often selected by physicians and pharmaceutics as the first line of treatment for various fungal infections that can occur in several body areas. 

Bachlav et al. studied novel micelle-based formulations, as an attempt to increase the bioavailability of azole antifungals, preparing CLZ, ECZ, and FLZ micelles with different copolymers, concluding that the optimal formulation can be achieved using ECZ loaded MPEG-dihexPLA, with an incorporation efficiency of 98.3% [46]. It was also demonstrated that the preparation method can influence the micelle properties, as faster evaporation and addition under sonication produced smaller PM. This PM based nanocarrier was incorporated in a gel pharmaceutical form and compared with the marketed liposomal formulation Pevaryl^®^. When applied to human and porcine skin, ECZ-PM achieved a significantly higher drug deposition in both ex vivo models. 

Elsalam et al. developed a mixed micelles composed of Pluronic P123 combined with Pluronic F127 and enriched with Cremophor EL as a stabilizer and penetration enhancer [47]. The purpose of this study was to assess the improvement of physical stability and skin delivery of TCZ in topical formulations. Following the ethanol injection method, several polymeric mixed micelle (PMM) formulations (F1-F8) were prepared, selecting different weight ratios of Pluronic P123 and Pluronic F127, as well as different percentages of Cremophor EL in the aqueous medium, obtaining a range of IE between 42.03 to 97.03%. Based on PMM size and IE profile, formulation F7 (40 weight ratio Pluronics/drug; 4 weight ratio Pluronic P123/Pluronic F127; and 5% Cremophor in aqueous solvent) was chosen as the optimal formulation and used in the subsequent studies.

**Figure 10:**
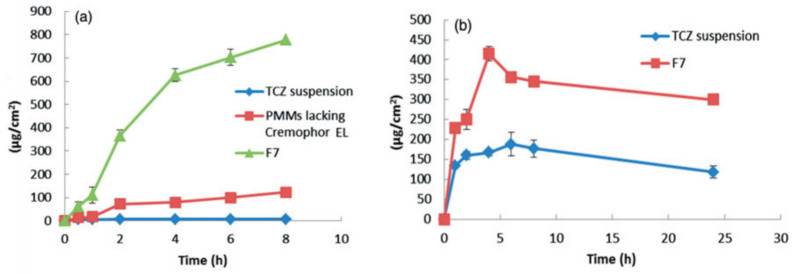
(**a**) Ex vivo cumulative amount of TCZ permeated per unit area across skin. (**b**) In vivo cumulative amount of TCZ deposited per unit area in the skin (adapted from [46]).

Ex vivo permeation study was performed in skin of newborn rats and showed that PMM when compared with the PMM formulation lacking Cremophor EL and TCZ suspension, the incorporation of surfactant in the PMM formulation enhanced the skin delivery (Figure 10) by potentially augmenting the interaction of PMM with the keratin in the corneocytes. In vivo, skin deposition of F7 was compared in TCZ suspension in mice, demonstrating a significantly higher calculated area under curve (AUC) through the 24 h-experiment, without showing inflammation, irritation, or significant alterations in the integrity of both the epidermis or the dermis layers of the skin, indicating the potential of PMM in overcoming the SC barrier and, thus, constituting a promising option to topical fungal infection treatments [46]. 

### 4.5. Other Skin Diseases and Conditions

#### 4.5.1. Imiquimod

One of the most prevalent malignancy in humans through the last decades is skin cancer, both melanoma and non-melanoma, including Bowen’s disease, basal cell carcinoma, and squamous cell carcinoma [60]. 

A non-invasive therapeutic option to skin cancer treatment is the topical application of imiquimod (IMQ), an imidazoquinoline that acts as an immunomodulator in antiviral and antitumoral potential in animal models. IMQ is poorly soluble in water and also shows little lipophilicity, constituting a challenge to its formulation in conventional topical formulations. Loaded nanomicelles were formulated by the solvent evaporation method, using methoxy-poly(ethylene glycol)-hexyl-substituted lactide (mPEG-hexPLA) as a copolymer, resulting in a micelles loaded with 0.05% IMQ. The gel formulation prepared from IMQ-loaded mPEG-hexPLA PMs in carboxymethyl cellulose was compared with the 5% IMQ marketed formulation Aldara^®^ and applied to both human and porcine skin The results showed that the IMQ-PM gel formulation outperformed Aldara^®^, presenting a higher delivery efficiency in viable epidermis and dermis [47]. 

#### 4.5.2. Spironolactone

Off-target over-activation of the mineralocorticoid receptor in chronic glucocorticoid therapy, especially in patients under specific conditions, such as diabetes, has been described as related to delays and complications in wound healing. Therefore, the combined administration of glucocorticoids with mineralocorticoid antagonists has been studied as a strategy to overcome this barrier, and has proven its efficacy studies conducted with mice. Spironolactone (SPL) is a non-selective mineralocorticoid receptor antagonist, also binding with progesterone and androgen receptors, and for that, its use off-label has been increasing in conditions such acne vulgaris, androgen alopecia, and hirsutism. However, there are not available topical formulations of SPL in the market, and its low water-solubility profile is once more a challenge to creating an easy-to-apply product [48,61]. 

Dahmana et al. formulated a 0.01% SPL (*w*/*w*) nanomicellar solution using mPEG-dihexPLA as the copolymer, and a 0.01% SPL (*w*/*w*) nanomicellar hydrogel, adding Carbopol^®^ to the previously prepared solution [49]. Since the aim of this study was to develop a formulation targeting the mineralocorticoids receptors (MR), it was mandatory to first demonstrate its presence and localization, and Dahmana et al. were able to prove, using immunofluorescence labelling, that MR are mostly located in the SPU and the sweat glands, and, thus, the targeted follicular delivery power of PM can also be of great usage in the case presented. After application of the developed nanomicellar formulations to PSU and PSU-free skin, the studies demonstrated that SPL was successfully delivered to the epidermis and upper dermis layers of the skin, with preferential delivery to the PSU, concluding that SPL can be applied topically in co-administration with glucocorticoids to prevent impaired wound healing. This nanoformulation could present an alternative to the oral administration of glucocorticoids in conditions involving androgen and progesterone receptors, such as acne vulgaris, androgen alopecia, and hirsutism, since the oral formulations of SPL can have a higher risk of systemic adverse effects. 

## 5. Nanotoxicology 

Nanoparticles occurred naturally in the environment over the course of the years and their intentional use dates from centuries, but their engineered production is relatively recent. Nanotechnology has been evolving over the last decades and offers several alternatives and solutions to existing problems in pharmaceutical and cosmetic product development and various other fields of application [40]. However, when working with submicron materials, safety issues and toxicity concerns towards human and environmental health can be raised, and it is required to assess the characterization of the nanocarrier materials, evaluating and optimizing their physicochemical properties, such as particle size, morphology, aspect ratio, and surface charge, that can influence the reactivity, transport, and nanotoxicity of the compounds.

When selecting polymers, biocompatible and biodegradable organic substances demonstrate lower toxicity levels than inorganic substances, as they minimize the risk of non-biodegradable foreign particles accumulation in the human body and the ecosystem. Furthermore, by-materials and sub-products of the nanosized constituents’ production can also exhibit potential toxicity and, thus, it is as fundamental to prudently ponder the proper production process of polymeric material and related nanosystems [58]. Nanocarriers can also add up to econanotoxicology and environmental concerns if an appropriate characterization and preparation is not conducted. Taking that into consideration, the major challenges include the assessment of the toxicology in complex ecosystems, the characterization of engineered nanomaterials in a natural environment, and the verification of the fate, possible accumulation and consequent effects in soil and the aquatic systems [62]. 

There are still some barriers in the translation of the in vitro to the in vivo PM toxicity results, hampering the total elimination of animal testing [63]. Kawaguchi et al. examined the toxicity of non-loaded PMs and even at significantly high doses, no pathological abnormality was found, observing only a transient activation of the mononuclear phagocytic system in the spleen and liver [64]. In the in vivo trials conducted with PM-based formulations, these nanocarrier systems proved a low toxicity profile, sometimes so mild that cannot be qualified as dose-limiting toxicity [65], especially when compared to other nanocarrier systems [66]. 

Despite these results, the nanotoxicity and econanotoxicity of PM-based carrier systems must be carefully assessed both in vitro and in vivo, and further guidance and regulation of the tests should be developed by the regulatory agencies [67]. 

## 6. Regulatory Aspects

The safety of the patient should be one of the main concerns when formulating a new pharmaceutical product containing nanomaterials. When regulating the research, development, and commercialization of nanomaterials in the world, the regulatory agencies lack in uniformizing the given information. For instance, nanomaterial-based nanomedicine can be classified as medicines and also as medical devices [67]. 

Nanomaterials are more difficult to categorize and characterize, and, given the number of parameters that need to be taken in consideration, the variety of nanomaterial constituents, models, and methodologies, this is a complex matter that failed a number of attempts to describe general rules [12]. In the EU and USA, the European Medicines Union (EMU) and the Food and Drug Administration (FDA)—the regulation bodies, respectively, have been publishing several preliminary guidelines for a range of nanomaterial-based medicine preparation standards. However, no formal document is presently published [68]. Furthermore, the prolonged process of development regarding nanomaterials can also be a complex matter when applying to a compound patent [68]. 

In a joint reflection paper issued by the EMU and EU Ministry for Health, Labour and Welfare *(Joint MHLW/EMA reflection paper on the development of block micelles medicinal products)* some of the challenges inherent to this fast-developing area of nanomedicine were addressed. The document provides basic information for the pharmaceutical development of the block-polymer nanomicelle drug products [69]. Although the outlined principles primarily focus on the products designed for intravenous administration, they can also be applied on other administration routes. It also recommends definition of the likely critical product attributes for each individual block polymer micelle formulation to account for the complexity of the nanosystem. In general, the critical quality attributes that could influence in vivo pharmacokinetics and dynamics, safety and efficacy should be defined. They include detailed physico–chemical description of the product (chemical composition, molecular weight, and polydispersity of block copolymer and its constituents, the composition of drug-loaded micelles; in vitro stability and drug release; in vivo copolymer degradation, osmolarity, site and release rate of active substance. Additionally, the impact of biologically active copolymer on the clinical efficacy and safety should also be evaluated. Since small changes in the composition of block copolymer micelles can influence their performance, a well-defined manufacturing process and process control are necessary to insure stable large-scale product manufacturing. Therefore, the quality control should include specifications of the starting materials, key intermediates and manufacturing impurities, production scale change, and process validation and evaluation. The application of Quality by Design is highly recommended to inure appropriate quality control. 

Due to the increasing incorporation of nanomaterials in cosmetic product formulations, it was necessary to create and implement specific legislation. To be commercialized in the European Union (EU) market, all cosmetic products must obey the Regulation European Commission (EC) no. 1223/2009 [70] and have a Product Information File (PIF) where, among other important information, a complete safety report has to be included [71]. 

Cosmetics containing nanomaterials in the formulation should be subjected to special requirements that need to be presented, such as the specification of the particle range size, and the declaration of the presence of nanomaterials in the product, as well as the use of the suffix ‘nano’, in brackets, after the name of the ingredient with nanomaterials, in the international nomenclature of cosmetic ingredients (INCI) list of ingredients [71]. Six months before the product enters the EU market, the responsible person—a legal person in the UE—has the duty to notify the nanomaterials in the products, online, in the EU Cosmetic Products Notification Portal (CPNP), where the information is accessible to Competent Authorities, Poison Centres, and cosmetic distributors [72]. Based on the existent information in the CPNP, the EC elaborated a Catalogue of Nanomaterials, containing all the nanomaterials approved for cosmetic use. In the USA, the FDA is the responsible agency and, unlike in the EU, cosmetic ingredients do not require the approval of the regulatory agency to be commercialized, excepting the colorant agents [73]. 

## 7. Concluding Remarks and Future Perspectives

Nanotechnology has become an instrument in all of its fields of application, proving its value in overcoming problems and reaching what in the past seemed unachievable. After decades of research on PM in the pharmaceutical science field, there are several clinical applications of PM-based products, and the future appears to be promising.

Considering the anatomy and physiology of human skin, the major challenge with pharmaceutical and cosmetic products is to successfully overcome the skin barrier, to penetrate more deeply in the skin layers and to sustain the release of the API to avoid overdosing that could result in possible adverse side effects and toxicity. In addition, drugs with high lipophilicity and low solubility are often more difficult to formulate and do not have high permeation. Formulations with APIs loaded in PMs for topical application can resolve that issue by loading these hydrophobic drugs in their inner core and make them, generally, more available to exert their therapeutic action, enhancing the efficacy even with lower doses per area unit and reducing, consequently, local irritation while preserving the integrity of the skin.

Reviewing the studies conducted over the last decade, it is possible to affirm that the incorporation of poorly soluble drugs in PM for topical treatment of skin diseases, such as acne vulgaris, psoriasis, and fungal infections, can be an opportunity for a successful therapeutic option, with a controlled release of the API, a higher bioavailability profile, higher efficacy and fewer local and systemic adverse side effects. Therefore, the perspective is that, in the years to come, more research and development is going to be made in the PM field, enlarging the applications and translating the in vivo results to more clinical trials and commercialized pharmaceutical products that suit the needs of the patients. 

In cosmetics, the competition and evolution of the market is notorious, and consumers are nowadays more informed regarding products and ingredients, demanding the finest galenic formulations and, therefore, continuously challenging the industry to overcome the barriers and deliver new and innovative solutions. Due to the increasing of life expectancy, the anti-ageing skincare market has grown over the years, with customers more interested in delaying the signs of the time, and the perspective is for this area to continue to evolve.

The future appears to be promising for PM-based formulations for dermal drug delivery, and proofs are given that PM might be one of the alternatives to conventional treatment options and conventional cosmetic products. Nevertheless, there is always room for further studies, and it is important to fully understand the consequences of the PM usage in larger scale in human health, the fauna, the flora and the complex ecosystems in our globe.

## Figures and Tables

**Table 1:** Polymer micelles prepared for skin delivery described in the literature in the last decade.

Active Compound	Structure	Polymers Used as Micellar Carriers	Conclusions	Ref.
	**ANTI-AGEING**			
Oleanolic Acid	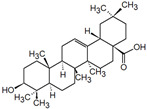	Poloxamer 407	Enhanced wrinkle alleviation	[39]
CoQ10	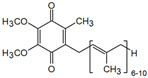	Oleyl-hyaluronan Hexyl-hyaluronan	Enhancement in skin hydration	[40]
	**ACNE VULGARIS**			
All-*trans* Retinoic Acid (Tretinoin)	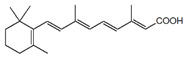	Poly(ethylene glycol) conjugated phosphatidylethanolamine	Higher stability with slower drug oxidation	[20]
All-*trans* Retinoic Acid (Tretinoin)		Diblock methoxy-poly(ethylene glycol)-poly(hexyl-substituted lactic acid)	Higher efficiency than marketed formulations	[41]
Adapalene	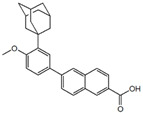	D-α-tocopheryl polyethyleneglycol 1000 succinate	Targeted drug delivery capacity Higher efficiency at lower dose than marketed formulations	[42]
Benzoyl peroxide	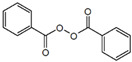	Pluronic^®^ F127	Targeted drug delivery capacity	[43]
	**PSORIASIS**			
Tacrolimus	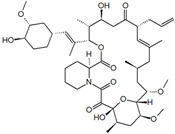	Diblock methoxy-poly(ethylene glycol)-poly(hexyl-substituted lactic acid)	Enhancement in skin drug deposition	[21]
Resveratrol	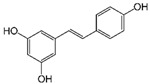	Pluronic^®^ P123 Pluronic^®^ F127	Decrease in the cytokine levels	[23]
Silibinin	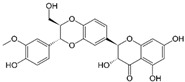	-	Reduction of psoriasis index area	[44]
	**FUNGAL INFECTIONS**			
Clotrimazole Fluconazole	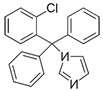 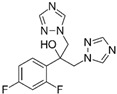	Diblock Methoxy-poly(ethylene glycol)-poly(hexyl-substituted lactic acid)	Enhancement in skin drug deposition	[45]
Terconazole	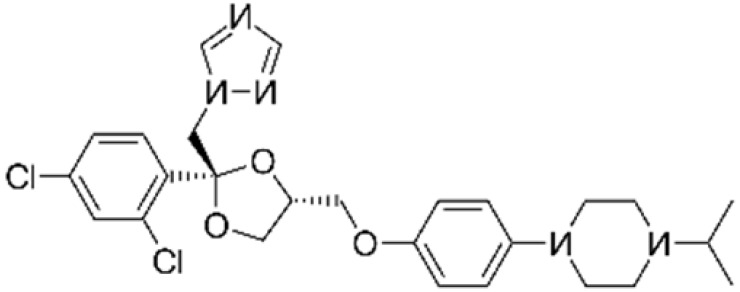	Pluronic^®^ P123 Pluronic^®^ F127 Cremophor EL	Higher permeation Higher skin deposition	[46]
	**OTHER SKIN DISEASES AND CONDITIONS**			
Imiquimod	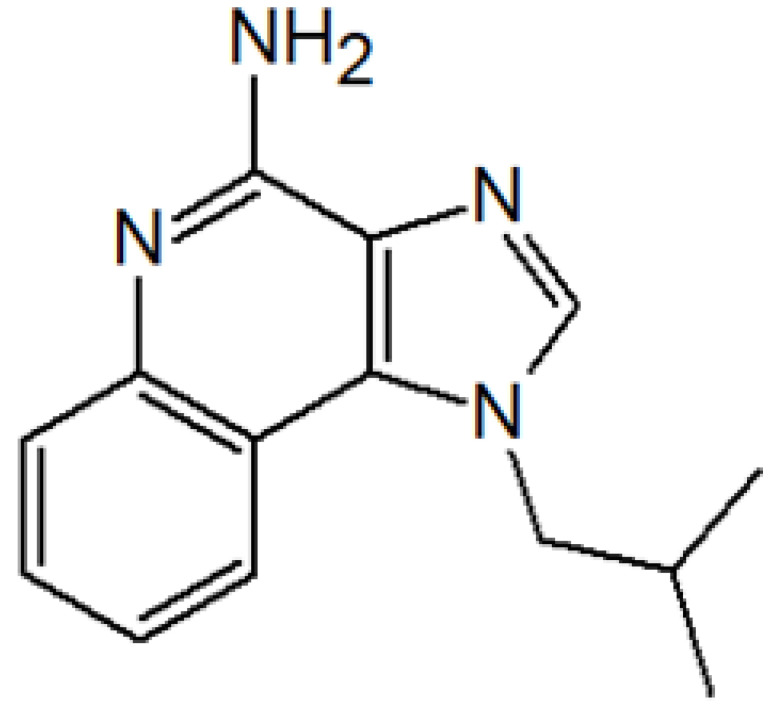	Diblock methoxy-poly(ethylene glycol)-)-hexyl-substituted lactide	Higher delivery efficiency at lower dose than the marketed formulation	[47]
Spironolactone	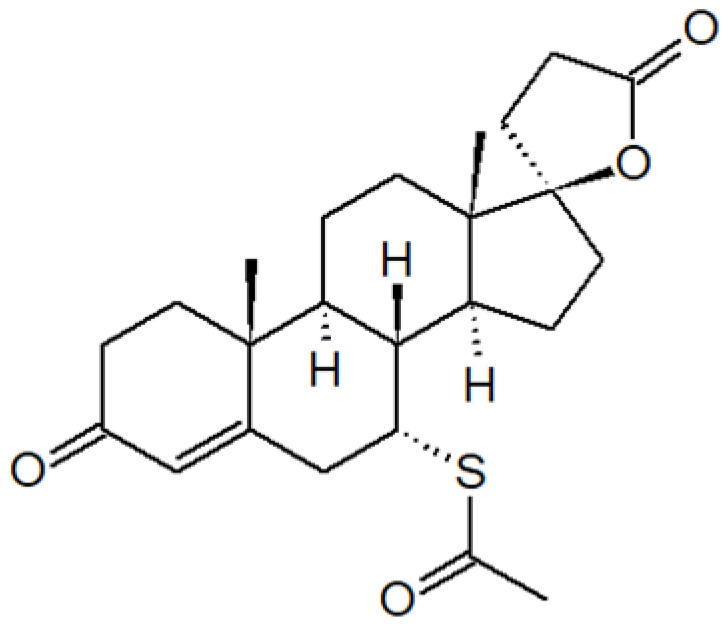	Diblock methoxy-poly(ethylene glycol)-poly(hexyl-substituted lactic acid)	Targeted drug delivery capacity	[48]

**Table 2:** Composition of micelles of oleanolic acid showing transparent liquid immediately after being diluted with distilled water [40].

Composition (*w/w*%)	PMO-G	PMO-H
Oleanolic Acid	0.05	0.05
Capryol^®^ 90	2	2
Poloxamer 407	6	7
